# Plant 4/1 protein: potential player in intracellular, cell-to-cell and long-distance signaling

**DOI:** 10.3389/fpls.2014.00026

**Published:** 2014-02-25

**Authors:** Sergey Y. Morozov, Svetlana S. Makarova, Tatyana N. Erokhina, Lilya Kopertekh, Joachim Schiemann, Robert A. Owens, Andrey G. Solovyev

**Affiliations:** ^1^A. N. Belozersky Institute of Physico-Chemical Biology, Moscow State UniversityMoscow, Russia; ^2^Department of Virology, Faculty of Biology, Moscow State UniversityMoscow, Russia; ^3^M. M. Shemyakin and Yu. A. Ovchinnikov Institute of Bioorganic Chemistry, Russian Academy of SciencesMoscow, Russia; ^4^Biosafety in Plant Biotechnology, Julius Kühn Institute – Federal Research Centre for Cultivated PlantsQuedlinburg, Germany; ^5^RetiredUSA

**Keywords:** intracellular transport, cell-to-cell transport, long-distance signaling, phloem transport, RNA binding protein

## Abstract

Originally isolated as a result of its ability to interact with the movement protein of *Tomato spotted wilt virus* in a yeast two-hybrid system, the 4/1 protein is proving to be an excellent tool for studying intracellular protein trafficking and intercellular communication. Expression of 4/1 *in vivo* is tightly regulated, first appearing in the veins of the cotyledon and later in the vasculature of the leaf and stem in association with the xylem parenchyma and phloem parenchyma. Structural studies indicate that 4/1 proteins contain as many as five coiled–coil (CC) domains; indeed, the highest level of sequence identity among 4/1 proteins involves their C-terminal CC domains, suggesting that protein–protein interaction is important for biological function. Recent data predict that the tertiary structure of this C-terminal CC domain is strikingly similar to that of yeast protein She2p; furthermore, like She2p, 4/1 protein exhibits RNA-binding activity, and mutational analysis has shown that the C-terminal CC domain is responsible for RNA binding. The 4/1 protein contains a nuclear export signal. Additional microscopy studies involving leptomycin and computer prediction suggest the presence of a nuclear localization signal as well.

## INTRODUCTION

Identification of host proteins involved in virus–host interactions has long been an area of intense interest for molecular virologists. Movement proteins (MPs) encoded by plant viruses were among the first viral proteins shown to have affinity for host components in experiments *in vivo* and *in vitro* (Citovsky et al., 1993; Wittmann et al., 1997; Dorokhov et al., 1999; Lazarowitz and Beachy, 2000; Harries et al., 2010).

To identify host factors that may play important roles in virus-specific processes, *Arabidopsis thaliana* and *Nicotiana* *benthamiana *cDNA libraries were screened in yeast two-hybrid system with NSm, the tubule-forming MP of *Tomato spotted wilt tospovirus *(TSWV), as bait (Soellick et al., 2000; von Bargen et al., 2001; Schoelz et al., 2011). Among the potentially interacting factors identified were a DnaJ-like co-chaperon (Soellick et al., 2000) and At-4/1, a previously uncharacterized protein showing some similarity to alpha-helical domains of myosin-, kinesin-, and ankyrin-like proteins (von Bargen et al., 2001). Although any functional relevance of At-4/1 for TSWV infection remains uncovered, subsequent studies have shown the 4/1 protein to be an excellent tool for studying intracellular protein trafficking and intercellular communication (Paape et al., 2006; Solovyev et al., 2013a).

## EVOLUTION OF THE 4/1 GENE

**FIGURE 1:**
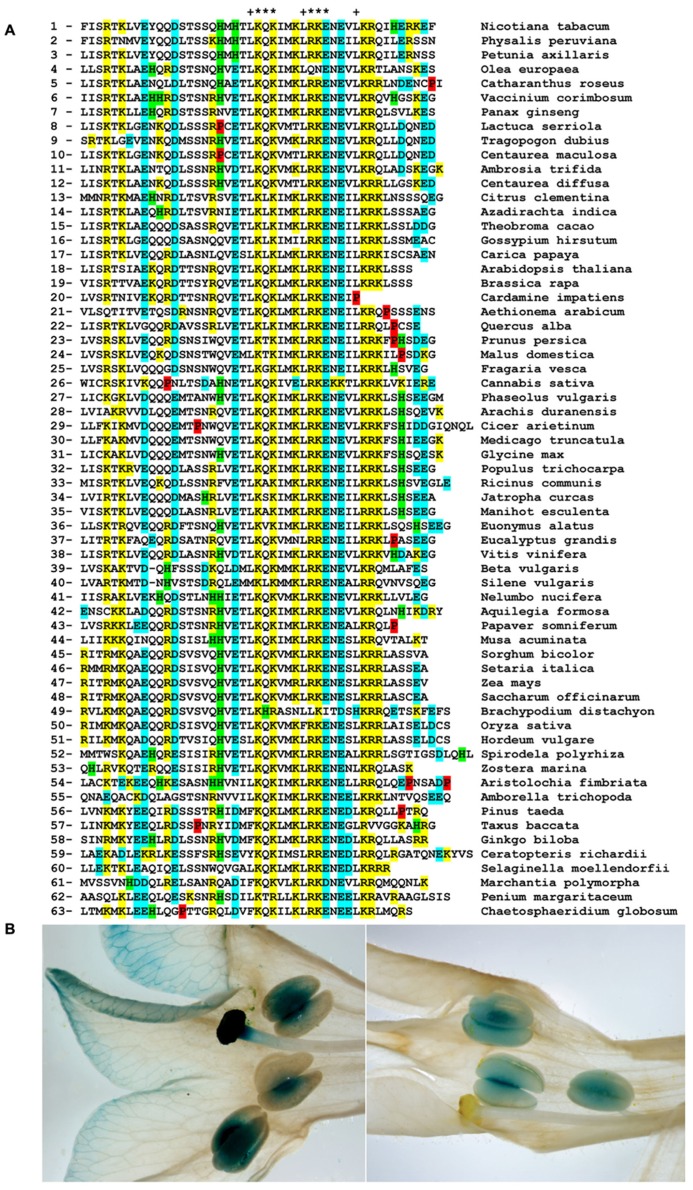
**(A)** Multiple amino acid sequence alignment of the C-terminal domains in 4/1 proteins. The conserved hydrophobic residues forming heptads (elements of potential leucine zippers) in 4/1 proteins are shown by (+) above the Nt-4/1 sequence. Residues replaced by alanines in Nt-4/1-KQK are marked with (*). Negatively charged residues are shown in blue, histidine residues are shaded in green, positively charged residues are shown in yellow, and prolines are shown in red. Taxonomic positions of the numbered plant species are as follows: 1-3 – **subclass asterids**, order Solanales; 4 – order Lamiales; 5 – order Gentianales; 6 – order Ericales; 7 – order Apiales; 8-12 – order Asterales; 13-14 – **subclass rosids**, order Sapindales; 15-16 – order Malvales; 17-21 – order Brassicales; 22 – order Fagales; 23-26 – order Rosales; 27-31 – order Fabales; 32-35 – order Malpighiales; 36 – order Celastrales; 37 – order Myrtales; 38 – order Vitales; 39-40 – **order Caryophyllales**; 41 – **order Proteales**; 42-43 – **order Ranunculales**; 44 – **class Liliopsida**, order Zingiberales; 45-51 – order Poales; 52-53 – order Alismatales; 54 – **order Piperales**; 55 – **order Amborellales**; 56-57 – **class Coniferopsida**, order Coniferales; 58 – **class Ginkgoopsida**, order Ginkgoales; 59 – **class Polypodiopsida**, order Polypodiales; 60 – **class Isoetopsida**, order Selaginellales; 61 – **class Marchantiopsida**, order Marchantiales; 62 – **class Zygnemophyceae**, order Desmidiales; 63 – **class Coleochaetophyceae**, order Coleochaetales. Please note that some species belonging to eudicotyledons (numbers 39–43) are not included into subclasses asterids and rosids; monocotyledons (class Liliopsida) are numbered as 44–53; plant species numbered as 54 and 55 are basal flowering plants. Species 62 and 63 represent microscopic algae. **(B)** Tissue-specific activity of the Nt-4/1 promoter in tobacco flowers. Promoter activity was analyzed in transgenic *Nicotiana tabacum *cv. Samsun plants carrying the GUS gene under the transcriptional control of either the 35S promoter used as a control (left panel), or a DNA fragment comprising 2000 base pairs upstream of the Nt-4/1 gene transcription start site (right panel).

4/1 genes encoded by the *A. thaliana *and *N. tabacum* genomes all contain eight exons and seven introns (Paape et al., 2006; Makarova et al., 2011). A similar exon–intron structure was found for most 4/1 genes encoded by other dicotyledonous and monocotyledonous plants. One remarkable exception was found in the order Rosales, where the 4/1 genes of all representatives sequenced (*Prunus persica*, *Malus domestica*, and *Fragaria vesca*) have lost introns 3 and 4 and thus contain only six exons (Makarova et al., 2011). **Figure 1A** shows an amino acid sequence alignment of the most conserved C-terminal region encoded by exon 8, which is predicted to form a coiled–coil (CC) structure (see below), for some of these proteins. In addition to flowering plants, 4/1 proteins are also found in gymnosperms (Coniferophyta and Ginkgophyta), ferns (Polypodiopsida, *Ceratopteris richardii*) and Lycopodiophyta (*Selaginella moellendorfii*; Makarova et al., 2011 and data not shown). It should be noted that in all known cases 4/1 represents a single-copy gene with an exception of *S.* *moellendorfii* and *N. tabacum* having two 4/1 gene copies due to polyploidy (Makarova et al., 2011).

Notably, we found clear evidence for the presence of a 4/1-like gene in Marchantiophyta (liverwort *Marchantia polymorpha*) that occupies a basal position among land plants (Bowman, 2013). The organization and encoded protein sequences of this putative ancestral 4/1 gene are similar to the five 3’-terminal exon–introns of the tobacco 4/1 gene (**Figure 1A** and our unpublished data). Moreover, our analysis of recently sequenced charophycean algae cDNA libraries (Timme et al., 2012) suggests that the typical C-terminal 4/1-like CC domains first appeared in orders Zygnematales (*Penium margaritaceum*) and Coleochaetales (*Chaetosphaeridium globosum*; **Figure 1A**). These algal orders are believed to represent the closest sister group to all land plants (Turmel et al., 2013). In contrast, no encoded 4/1-like protein signatures were found in moss *Physcomitrella patens*.

## TISSUE- AND ORGAN-SPECIFIC EXPRESSION OF 4/1 PROTEIN

To analyze the tissue specificity and developmental regulation of Nt-4/1 gene expression, we generated transgenic tobacco and *N. benthamiana* plants carrying the GUS reporter gene under the control of Nt-4/1 promoter (Solovyev et al., 2013b). Our analyses revealed that 4/1 promoter-driven expression was first observed in cotyledons in association with veins. Later, at the two- to four-leaf stage, GUS staining was found in veins in the blades of first two foliage leaves as well as in cotyledon veins. As the plants continued to grow, GUS staining disappeared from the cotyledons and was found in association with veins of younger leaves and stem internodes, with the hypocotyl being the most intensively stained. Studies of tissue- and cell-specific expression in these organs showed that in larger veins showing secondary growth, staining was associated mostly with the xylem parenchyma and phloem parenchyma. In young veins without secondary growth staining was limited to primary phloem cells. Stems and mature leaves showed GUS expression in vascular bundles, mostly in xylem parenchyma (Solovyev et al., 2013b).

Edwards et al. (2010) have used Affymetrix GeneChip arrays to study tobacco gene expression from seed germination to senescence. Among the 19 different tobacco organ samples tested, 4/1 gene expression was highest in vegetative and floral shoot apices, floral buds, and juvenile leaves. Somewhat lower levels of expression were observed in stem internodes, seeds, and cotyledons (Edwards et al., 2010). Thus, the Affymetrix data sets for tobacco are in good agreement with the results of our GUS expression experiments.

Detailed information on the organ-, or tissue-dependent expression pattern of plant genes can be retrieved from several public domain microarray databases, e.g., AtGenExpress at , PLEXdb at , eFP browser at  and NCBI GEO at . In poplar, 4/1 expression was very high in differentiating xylem (NCBI GEO GSE30507, GSE25309, GSE25304; Wilkins et al., 2009). In growing tips of maize leaves, the highest levels of 4/1 mRNA were present in the bundle sheath of developing veins (Li et al., 2010), whereas according to the Genevestigator database () significant expression levels were observed in the phloem. Second, similar to the pattern observed in tobacco, extremely high levels of 4/1 mRNA were found in the shoot apex (especially the apical meristem) of *A. thaliana* (Schmid et al., 2005), soybean (Libault et al., 2010), and rice (Jain et al., 2007). Third, a significant increase in 4/1 mRNA expression was observed at certain stages of seed formation in *A. thaliana *(most prominently in chalazal endosperm; Genevestigator and ), *Medicago truncatula* (He et al., 2009), soybean (Severin et al., 2010), and rice (Jain et al., 2007). These findings indicate that in addition to the conservation of gene structure and sequence of the encoded proteins, the patterns of 4/1 gene expression in evolutionary distant species are quite similar. Notably, according to the Genevestigator database the highest levels of 4/1 mRNA were present in pollen of *A. thaliana *(gene At4g26020) and in anther/pollen of *Oryza sativa* (gene Os02g0148600). In agreement with these data, we found 4/1 promoter activity in anthers, but not other parts of *N. tabacum *flowers, whereas the 35S promoter used as a control was active in pistils, anthers and petal veins (**Figure 1B**).

## PHYSICO-CHEMICAL PROPERTIES OF 4/1 PROTEIN

The Nt-4/1 protein is predicted by PSSFinder (Soli et al., 2009) () to form six long alpha-helices and two short beta-sheet structures. PCOIL (Lupas, 1996; Gruber et al., 2006; ) and MARCOIL (Delorenzi and Speed, 2002;  webmarcoil/webmarcoilC1.html) predicted the likely presence of five CC domains in the alpha-helical regions (positions 44-71, 115-131, 139-167, 176-209, and 223-243; Makarova et al., 2011). Similar secondary structure elements were also predicted for other 4/1 proteins (see, for example **Figure 1A**). In many proteins, CC domains regulate homo- and hetero-multimerization important for the protein function (Gordon et al., 2009; Wang and Coluccio, 2010). Interestingly, the highest level of sequence identity among all 4/1 proteins is found between the C-terminal CC domains (Paape et al., 2006; **Figure 1A**).

To experimentally analyse the secondary structure of the Nt-4/1 protein, the circular dichroism spectra in the far UV region were measured for wild type and several mutant Nt-4/1 proteins. The α-helix content in these polypeptides was estimated using the Greenfield–Fasman equation (Greenfield and Fasman, 1969). This calculation gave a value of 43% for the wild type protein (Makarova et al., 2011). Partial proteolysis data and differential scanning calorimetry (DSC) indicated that the Nt-4/1 protein consists of three structural domains: an N-terminal domain with a relatively significant level of disorder, a more stable central domain capable of interacting with the N-terminal part and the most stable C-terminal region that forms the 5th CC domain represented in **Figure 1A**.

## INTERACTION WITH NUCLEIC ACIDS

**FIGURE 2:**
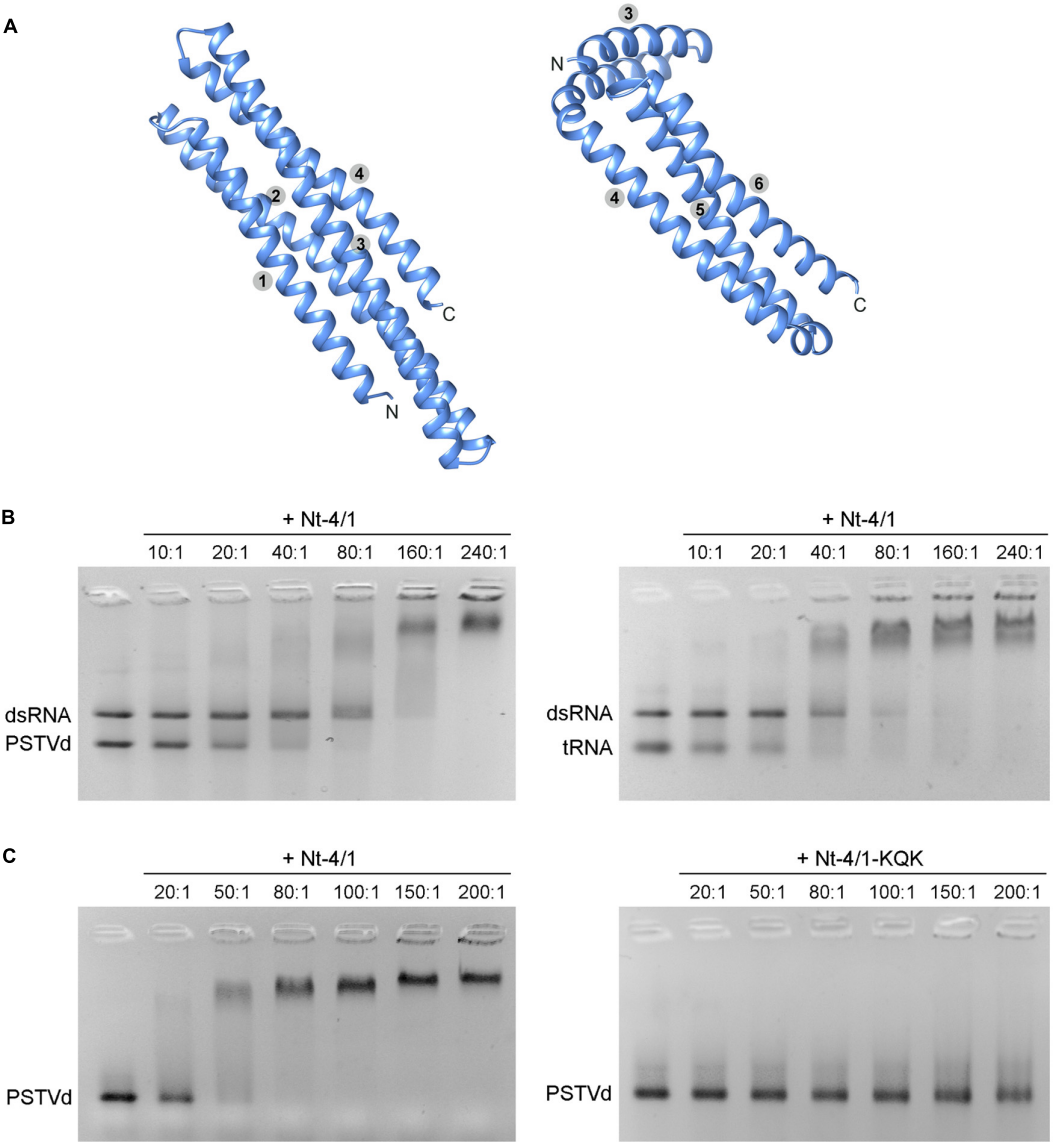
**(A)** QUARK predictions of 4/1 structure. Predictions for the *M. polymorpha* 4/1 protein possessing four alpha helices (left panel) and an Nt-4/1 segment including four C-terminal alpha helices (right panel) are shown. Numbers indicate positions of alpha helices in the entire proteins. Graphical presentation of predicted structures was performed with the UCSF Chimera package (Pettersen et al., 2004). **(B) **Gel retardation analysis of the competitive ability of Nt-4/1 to binding viroid RNA, tRNA and dsGFP RNA. Increasing amounts of Nt-4/1 (molar ratios of protein:RNA ratios are indicated above each lane) were incubated with RNA prior to gel loading. **(C)** Comparison of viroid RNA binding by Nt-4/1 and the mutant Nt-4/1-KQK (see **Figure 1A**).

To further investigate the spatial organization of 4/1 protein, we used a new algorithm, QUARK (), developed for *ab initio* protein structure prediction. For a query protein sequence, this algorithm first predicts a variety of selected structural features using a neural network. The global fold is then generated by replica-exchange Monte Carlo simulations which assemble the small fragments as generated by gapless threading through the template library (Xu and Zhang, 2012). Because of a length limit of 199 residues for the query sequence, we used QUARK to compare the organization of the shortest 4/1 protein from *M. polymorpha* (196 amino acids) and an Nt-4/1 deletion mutant lacking the N-terminal 90 amino acids with two predicted alpha helices (**Figure 2A**). Both proteins exhibited very similar tertiary structures, mainly an alpha up-down bundle fold.

Next, we used the COFACTOR webserver (Roy et al., 2012) () for automated structure-based protein function annotation. Starting from a structural model, derived from either experimental determination or computational modeling, COFACTOR identifies template proteins of similar folds. Quite unexpectedly, 4/1 proteins showed the greatest similarity in their folding to yeast protein She2p (246 amino acids in length). This protein is a member of a class of nucleic acid binding proteins that contain a single globular domain with a five alpha helix bundle and form a symmetric homodimer (Niessing et al., 2004; **Figure 2A**). In yeast, this protein is involved in directional transport and localization of a specific mRNA (*ASH1* mRNA) that regulates gene expression on a temporal and spatial level (Jansen and Niessing, 2012). During mitosis in *Saccharomyces cerevisiae*, *ASH1* mRNA is transported from the mother cell to the daughter cell as part of a large mRNP which contains She2p. *ASH1* mRNA contains four *cis*-acting hairpin regions, termed zip-code elements, that mediate mRNA binding to She2p as well as its subsequent directed intracellular transport (Jansen and Niessing, 2012).

Because we had previously observed homo-oligomerization of At-4/1 in the yeast two-hybrid assay (Minina et al., 2009), we were interested to determine whether or not Nt-4/1 was also capable of forming oligomers. Preparations of recombinant Nt-4/1 were analyzed by dynamic laser light scattering, a method that measures the size distribution of small particles dispersed in solution, and electron microscopy. The resulting data indicated that Nt-4/1 exists in solution predominantly as oligomers and multimers (Solovyev et al., 2013a).

To experimentally investigate possible functional similarities between She2p and 4/1 proteins, the ability of Nt-4/1 protein to bind various forms of RNA was investigated using a combination of agarose gel retardation and Northwestern assays. First, Nt-4/1 was shown to bind single-stranded green fluorescent protein (GFP) transcripts much more weakly than double-stranded RNA obtained by annealing with a complementary RNA chain (data not shown). Interestingly, Potato spindle tuber viroid (PSTVd; Gross et al., 1978) which is an imperfect dsRNA (thus showing a similarity to zip-code elements in *ASH1* mRNA) binds Nt-4/1 more strongly than non-specific GFP dsRNA (**Figure 2B**). Likewise, partially double-stranded tRNA interacts with Nt-4/1 significantly better than non-specific GFP dsRNA (**Figure 2B**). Deletion of the C-terminal CC domain in Nt-4/1 mutant ΔCCV blocked viroid binding (to be presented eslewhere). These data suggest that the positively charged and highly conserved C-terminal region of 4/1 (**Figure 1A**) plays an important role in RNA binding. To verify this hypothesis, we constructed a Nt-4/1 mutant called Nt-4/1-KQK in which six amino acid residues in the C-terminal CC region including three Lys and one Arg residue were replaced with Ala residues (**Figure 1A**). Similar to Nt-4/1-ΔCCV, Nt-4/1-KQK was unable to interact with PSTVd (**Figure 2C**). Collectively, the structural predictions and data on protein multimerization and RNA binding indicate that Nt-4/1 protein and yeast She2p share several common features, but further research is required to determine whether or not these similarities reflect a general mode of action involving binding of imperfect RNA duplexes. It is tempting to speculate that 4/1 protein, similarly to She 2p, might be involved in addressed transport of RNA molecules to specific subcellular compartments in plants. Taking into account the fact that the 4/1 expression is associated with the vascular tissues, it seems likely that this protein is involved in the long-distance RNA transport through the phloem.

Indeed, we have recently reported evidence for a possible role of 4/1 protein in the long-distance vascular movement of PSTVd (Solovyev et al., 2013a). When PSTVd was inoculated onto *N. benthamiana *plants where the level of endogenous 4/1 mRNA was down-regulated by virus-induced gene silencing (VIGS), long-distance movement of the viroid into developing young leaves above the inoculated leaf was much more efficient than in comparable unsilenced control plants (Solovyev et al., 2013a). These data raise another important question for further studies, namely, does the interaction of 4/1 with the viroid contributes to long-distance pathogen movement or, alternatively, to its restriction?

## SUBCELLULAR LOCALIZATION OF 4/1 PROTEIN

Thus far we have generated two sets of experimental data concerning the subcellular localization of 4/1 protein. First, At-4/1 fused at its C-terminus to GFP, and the corresponding expression vectors were used in particle bombardment assays (Paape et al., 2006). Confocal laser scanning microscopy (CLSM) revealed that a majority of At-4/1 assembled in punctate bodies at the cell periphery. For simplicity, the 4/1-specific structures were called ‘FO bodies’ (four/one bodies; Solovyev et al., 2013a). A striking feature of At-4/1-GFP was the polarized distribution of FO bodies in epidermal cells of *A. thaliana *and *N. benthamiana *leaves. The protein was localized predominantly in one-half of an epidermal cell. The molecular basis for this peculiar subcellular distribution is currently unknown, but among several signaling pathways and networks that regulate protein intracellular polarity in plants (Yang, 2008) the PIN auxin efflux carriers (Barbez and Kleine-Vehn, 2013; Moschou et al., 2013) seem the most likely candidates for future comparative studies with 4/1.

In many cases, the At-4/1 FO bodies located at the cellular periphery appeared as twin structures consisting of two disconnected bodies. Superposition of confocal and bright field images showed that twin FO bodies are located on opposite sides of the cell walls separating adjacent leaf epidermal cells. Twin-body formation may reflect the association of FO bodies at opposite neck regions of a particular PD or within the same pit field (Paape et al., 2006). Because the At-4/1-GFP fusion protein was expressed via particle bombardment of single isolated cells, observations of twin bodies in these experiments implies that At-4/1 is able to move through plasmodesmata between cells (Paape et al., 2006). In the second series of experiments, the subcellular localization of Nt-4/1 GFP fusion protein was analyzed by agroinfiltration-mediated transient expression (Solovyev et al., 2013a). Although most of the Nt-4/1 FO bodies behaved as stationary structures, some of them moved within the cell. This intracellular movement of FO bodies depended on the actin-myosin system. Cytochalasin D, known to disassemble microfilaments and interfere with the actin-dependent protein trafficking in plant cells, blocked movement of these FO bodies. When we co-expressed Nt-4/1-GFP and YFP-talin, a marker for the actin cytoskeleton, most of the FO bodies co-aligned with the actin microfilaments (Solovyev et al., 2013a). Interestingly, yeast two-hybrid analysis revealed that At-4/1 is also capable of binding plant actin 7 (Minina et al., 2009 and unpublished data).

Previously, we have hypothesized that the Nt-4/1 protein is capable of shuttling between the nucleus and cytoplasm (Minina et al., 2009). To test this hypothesis, cells expressing Nt-4/1-GFP were treated with leptomycin B, a drug known to suppress the function of CRM1/exportin 1, a carrier protein involved in nuclear export of proteins containing a nuclear export signal (NES). In this case, granular fluorescent structures were observed within the nuclei as well as the cytoplasm of leptomycin B-treated cells (Solovyev et al., 2013a). The ability of Nt-4/1 protein to localize both to the nucleus and the cytoplasm as well as the effect of leptomycin B on its localization implies that this protein possesses both a nuclear localization signal (NLS) and a NES. Indeed, mutational analysis of Nt-4/1 has allowed us to identify a single NES (residues 196-205 of the Nt-4/1 sequence). A search for non-canonical NLSs in Nt-4/1 protein using “cNLS Mapper” () predicted three medium-score NLSs located at residues 40-68 (NLS1), 150-183 (NLS2), and 202-233 (NLS3) of the Nt-4/1 sequence (Solovyev et al., 2013a).

Mutation of the NES (Nt-4/1 CCII mutant) resulted in accumulation of Nt-4/1 within the nucleoplasm. The mutant protein accumulated as large spherical bodies as much as 4 nm in diameter. The majority of nuclei observed contained either one or two Nt-4/1-CCII bodies. Single optical sections obtained by CLSM demonstrated that Nt-4/1-CCII-GFP was localized only at the periphery of the spherical structures. Immunogold labeling confirmed that Nt-4/1-CCII-GFP was localized only to the peripheral electron-dense layer of the nuclear bodies. The internal regions contained moderately electron-dense material, suggesting that proteins other than Nt-4/1-CCII-GFP could be located in the interior of the spherical structures. The question remains how do (and if) potential accessory proteins influence formation of large nuclear bodies in Nt-4/1 NES mutant? To this end, experiments to isolate the large *N. benthamiana* nuclear 4/1 bodies and determine their molecular composition are currently in progress.

## FUTURE PERSPECTIVES

In this brief review, we have attempted to provide an overall picture of what is currently known about 4/1 protein. However, as additional evidence continues to accumulate, new and compelling questions arise, questions that must be answered in the next years if we are to have a more complete and realistic view of the properties of 4/1. First, comparison of the varying patterns of 4/1 gene expression (or complete lack thereof) in different plant species may help to reconstruct the molecular networks where this gene is involved. To this end, we plan to create *N. benthamiana* 4/1 knock-down plants and, additionally, potato and tomato plants expressing heterologous 4/1 proteins. To our surprise BLAST analysis of sequence data from solgenomics.net indicates that both these crops (like tobacco, members of the Solanaceae) appear to lack 4/1 genes. This notion can be due to the incompleteness of available genome sequences. However, potato and tomato are natural hosts for PSTVd. This may correlate with both the lack of 4/1 in these plant and enhanced PSTVd long-distance movement in 4/1-silenced *N. benthamiana* (Solovyev et al., 2013b). Second, in the last two decades, many viral MP determinants that are involved in systemic invasion of plants have been identified and characterized. The current state of the art directs us toward the following questions: how are MP-4/1 complexes formed? Once formed, how are these complexes distributed in the plant cell? Given the unanticipated diversity of MP types and assuming that 4/1 protein interacts with the tubule-forming MPs of nepo- as well as tospoviruses (von Bargen, personal communication), it will be necessary to study the molecular dynamics of complex formation in a variety of MP contexts to unveil the spectrum of different mechanisms regulating MP activities after 4/1 binding.

## Conflict of Interest Statement

The authors declare that the research was conducted in the absence of any commercial or financial relationships that could be construed as a potential conflict of interest.

## References

[B1] Barbez E. Kleine-Vehn J. (2013). Divide Et Impera - cellular auxin compartmentalization. *Curr. Opin. Plant Biol.* 16 78–84 10.1016/j.pbi.2012.10.005 23200033

[B2] Bowman J. L. (2013). Walkabout on the long branches of plant evolution. *Curr. Opin. Plant Biol.* 16 70–77 10.1016/j.pbi.2012.10.001 23140608

[B3] Citovsky V. McLean B. G. Zupan J. R. Zambryski P. (1993). Phosphorylation of tobacco mosaic virus cell-to-cell movement protein by a developmentally regulated plant cell wall-associated protein kinase. *Genes Dev.* 7 904–910 10.1101/gad.7.5.904 7684009

[B4] Delorenzi M. Speed T. (2002). An HMM model for coiled-coil domains and a comparison with PSSM-based predictions. *Bioinformatics* 18 617–625 10.1093/bioinformatics/18.4.617 12016059

[B5] Dorokhov Y. L. Mäkinen K. Frolova O. Y. Merits A. Saarinen J. Kalkkinen N. (1999). A novel function for a ubiquitous plant enzyme pectin methylesterase: the host-cell receptor for the tobacco mosaic virus movement protein. *FEBS Lett.* 461 223–228 10.1016/S0014-5793(99)01447-7 10567701

[B6] Edwards K. D. Bombarely A. Story G. W. Allen F. Mueller L. A. Coates S. A. (2010). TobEA: an atlas of tobacco gene expression from seed to senescence. *BMC Genomics* 11:142 10.1186/1471-2164-11-142 PMC2841117 20187945

[B7] Gordon D. E. Mirza M. Sahlender D. A. Jakovleska J. Peden A. A. (2009). Coiled-coil interactions are required for post-Golgi R-SNARE trafficking. *EMBO Rep.* 10 851–856 10.1038/embor.2009.96 19557002 PMC2726663

[B8] Greenfield N. Fasman G. D. (1969). Computed circular dichroism spectra for the evaluation of protein conformation. *Biochemistry* 8 4108–4116 10.1021/bi00838a031 5346390

[B9] Gross H. J. Domdey H. Lossow C. Jank P. Raba M. Alberty H. (1978). Nucleotide sequence and secondary structure of potato spindle tuber viroid. *Nature* 273 203–208 10.1038/273203a0 643081

[B10] Gruber M. Söding J. Lupas A. N. (2006). Comparative analysis of coiled-coil prediction methods. *J. Struct. Biol.* 155 140–145 10.1016/j.jsb.2006.03.009 16870472

[B11] Harries P. A. Schoelz J. E. Nelson R. S. (2010). Intracellular transport of viruses and their components: utilizing the cytoskeleton and membrane highways. *Mol. Plant Microbe Interact.* 11 1381–1393 10.1094/MPMI-05-10-0121 20653412

[B12] He J. Benedito V. A. Wang M. Murray J. D. Zhao P. X. Tang Y. (2009). The *Medicago truncatula* gene expression atlas web server. *BMC Bioinformatics* 10:441 10.1186/1471-2105-10-441 PMC2804685 20028527

[B13] Jain M. Nijhawan A. Arora R. Agarwal P. Ray S. Sharma P. (2007). F-box proteins in rice. Genome-wide analysis, classification, temporal and spatial gene expression during panicle and seed development, and regulation by light and abiotic stress.* Plant Physiol.* 143 1467–1483 10.1104/pp.106.091900 PMC1851844 17293439

[B14] Jansen R. P. Niessing D. (2012). Assembly of mRNA-protein complexes for directional mRNA transport in eukaryotes–an overview. *Curr. Protein Pept. Sci.* 13 284–293 10.2174/138920312801619493 22708485 PMC3474952

[B15] Lazarowitz S. G. Beachy R. N. (2000). Viral movement proteins as probes for intracellular and intercellular trafficking in plants. *Plant Cell* 11 535–548 10213776 10.1105/tpc.11.4.535 PMC144200

[B16] Li P. Ponnala L. Gandotra N. Wang L. Si Y. Tausta S. L. (2010). The developmental dynamics of the maize leaf transcriptome. *Nat. Genet.* 42 1060–1067 10.1038/ng.703 21037569

[B17] Libault M. Farmer A. Joshi T. Takahashi K. Langley R. J. Franklin L. D. (2010). An integrated transcriptome atlas of the crop model Glycine max, and its use in comparative analyses in plants. *Plant J.* 63 86–99 10.1111/j.1365-313X.2010.04222.x 20408999

[B18] Lupas A. (1996). Coiled coils: new structures and new functions. *Trends Biochem. Sci.* 21 375–382 10.1016/S0968-0004(96)10052-9 8918191

[B19] Makarova S.S. Minina E.A. Makarov V.V. Semenyuk P.I. Kopertekh L. Schiemann J. (2011). Orthologues of a plant-specific At-4/1 gene in the genus *Nicotiana* and the structural properties of bacterially expressed 4/1 protein. *Biochimie* 93 1770–1778 10.1016/j.biochi.2011.06.018 21712068

[B20] Minina E.A. Erokhina T.N. Garushyants S.K. Solovyev A. G. Morozov S. Y. (2009). Subcellular localization of the new plant protein 4/1 and analysis of heterologous protein-protein interactions indicate its ability for nuclear-cytoplasmic transport. *Dokl. Biochem. Biophys.* 429 296–300 10.1134/S1607672909060039 20101824

[B21] Moschou P. N. Smertenko A. P. Minina E. A. Fukada K. Savenkov E. I. Robert S. (2013). The caspase-related protease separase (extra spindle poles) regulates cell polarity and cytokinesis in *Arabidopsis*. *Plant Cell* 25 2171–2186 10.1105/tpc.113.113043 23898031 PMC3723619

[B22] Niessing D. Hüttelmaier S. Zenklusen D. Singer R. H. Burley S. K. (2004). She2p is a novel RNA binding protein with a basic helical hairpin motif. *Cell* 119 491–502 10.1016/j.cell.2004.10.018 15537539

[B23] Paape M. Solovyev A. G. Erokhina T. N. Minina E. A. Schepetilnikov M. V. Lesemann D. E. (2006). At-4/1, an interactor of the tomato spotted wilt virus movement protein, belongs to a new family of plant proteins capable of directed intra- and intercellular trafficking. *Mol. Plant Microbe Interact.* 19 874–883 10.1094/MPMI-19-0874 16903353

[B24] Pettersen E. F. Goddard T. D. Huang C. C. Couch G. S. Greenblatt D. M. Meng E. C. (2004). UCSF Chimera - a visualization system for exploratory research and analysis. *J. Comput. Chem.* 25 1605–1612 10.1002/jcc.20084 15264254

[B25] Roy A. Yang J. Zhang Y. (2012). COFACTOR: an accurate comparative algorithm for structure-based protein function annotation. *Nucleic Acids Res. *40 (Web Server issue) W471–W477 10.1093/nar/gks372 PMC3394312 22570420

[B26] Schmid M. Davison T. S. Henz S. R. Pape U. J. Demar M. Vingron M. (2005). A gene expression map of *Arabidopsis thaliana* development. *Nat. Genet.* 37 501–506 10.1038/ng1543 15806101

[B27] Schoelz J. E. Harries P. A. Nelson R. S. (2011). Intracellular transport of plant viruses: finding the door out of the cell. *Mol. Plant* 4 813–831 10.1093/mp/ssr070 21896501 PMC3183398

[B28] Severin A. J. Woody J. L. Bolon Y. T. Joseph B. Diers B. W. Farmer A. D. (2010). RNA-Seq atlas of glycine max: a guide to the soybean transcriptome. *BMC Plant Biol.* 10:160 10.1186/1471-2229-10-160 PMC3017786 20687943

[B29] Soellick T. Uhrig J. F. Bucher G. L. Kellmann J. W. Schreier P. H. (2000). The movement protein NSm of tomato spotted wilt tospovirus (TSWV): RNA binding, interaction with the TSWV N protein, and identification of interacting plant proteins. *Proc. Natl. Acad. Sci. U.S.A.* 97 2373–2378 10.1073/pnas.030548397 10688879 PMC15808

[B30] Soli R. Kaabi B. Barhoumi M. El-Ayeb M. Srairi-Abid N. (2009). Bioinformatic characterizations and prediction of K+ and N+ ion channels effector toxins. *BMC Pharmacol*. 9:4–11 10.1186/1471-2210-9-4 19284552 PMC2660317

[B31] Solovyev A. G. Minina E. A. Makarova S. S. Erokhina T. N. Makarov V. V. Kaplan I. B. (2013a). Subcellular localization and self-interaction of plant-specific Nt-4/1 protein. *Biochimie* 95 1360–1370 10.1016/j.biochi.2013.02.015 23499290

[B32] Solovyev A. G. Makarova S. S. Remizowa M. V. Lim H. S. Hammond J. Owens R. A. (2013b). Possible role of the Nt-4/1 protein in macromolecular transport in vascular tissue. *Plant Signal. Behav*. 8:10 10.4161/psb.25784 PMC4091084 23887490

[B33] Timme R. E. Bachvaroff T. R. Delwiche C. F. (2012). Broad phylogenomic sampling and the sister lineage of land plants. *PLoS ONE* 7:e29696 10.1371/journal.pone.0029696 PMC3258253 22253761

[B34] Turmel M. Otis C. Lemieux C. (2013). Tracing the evolution of streptophyte algae and their mitochondrial genome. *Genome Biol. Evol*. 5 1817–1835 10.1093/gbe/evt135 24022472 PMC3814193

[B35] von Bargen S. Salchert K. Paape M. Piechulla B. Kellmann J.-W. (2001). Interactions between the tomato spotted wilt virus movement protein and plant proteins showing homologies to myosin, kinesin and DnaJ-like chaperones. *Plant Physiol. Biochem.* 39 1083–1093 10.1016/S0981-9428(01)01331-6

[B36] Wang C. L. Coluccio L. M. (2010). New insights into the regulation of the actin cytoskeleton by tropomyosin. *Int. Rev. Cell Mol. Biol.* 281 91–128 10.1016/S1937-6448(10)81003-2 20460184 PMC2923581

[B37] Wilkins O. Nahal H. Foong J. Provart N. J. Campbell M. M. (2009). Expansion and diversification of the Populus R2R3-MYB family of transcription factors. *Plant Physiol.* 149 981–993 10.1104/pp.108.132795 19091872 PMC2633813

[B38] Wittmann S. Chatel H. Fortin M. G. Laliberté J. F. (1997). Interaction of the viral protein genome linked of turnip mosaic potyvirus with the translational eukaryotic initiation factor (iso) 4E of *Arabidopsis thaliana* using the yeast two-hybrid system. *Virology* 234 84–92 10.1006/viro.1997.8634 9234949

[B39] Xu D. Zhang Y. (2012). Ab initio protein structure assembly using continuous structure fragments and optimized knowledge-based force field. *Proteins* 80 1715–1735 10.1002/prot.24065 22411565 PMC3370074

[B40] Yang Z. (2008). Cell polarity signaling in *Arabidopsis*. *Annu. Rev. Cell Dev. Biol.* 24 551–575 10.1146/annurev.cellbio.23.090506.123233 18837672 PMC2739732

